# Whole‐exome sequencing identified novel variants in *CPLANE1* that causes oral‐facial‐digital syndrome Ⅵ by inducing primary cilia abnormality

**DOI:** 10.1111/jcmm.17326

**Published:** 2022-05-18

**Authors:** Wen Qian, Xinlei Liu, Zhengrong Wang, Yongjie Xu, Jingzhi Zhang, Haizhi Li, Qiang Zhong, Chengcheng Li, Liying Zhu, Zunlun Zhou, Wei Pan

**Affiliations:** ^1^ Prenatal Diagnosis Center in Guizhou Province The Affiliated Hospital of Guizhou Medical University Guiyang China; ^2^ 74628 School of Medical Laboratory Guizhou Medical University Guiyang China; ^3^ 74628 School of Public Health Key Laboratory of Environmental Pollution Monitoring and Disease Control Guizhou Medical University Guiyang China; ^4^ 74628 Department of Obstetrics and Gynecology The Affiliated Hospital of Guizhou Medical University Guiyang China

## Abstract

Oral‐facial‐digital syndrome (OFDS) is a multisystemic ciliopathic disorder with an autosomal recessive mode of inheritance. OFDS usually manifests with typical craniofacial anomalies and variable occurrence of polydactyly. Germline variants in *CPLANE1* cause OFDS VI. In this study, we investigated a 26‐year‐old Chinese female patient who was 23^+1^ weeks pregnant. She had a history of adverse pregnancy outcomes with multiple foetal malformations. We performed ultrasonography and identified the foetus as having a posterior fossa Blake cyst and postaxial polydactyly. The patient decided to terminate her pregnancy, and further genetic molecular analysis was performed. We identified the aborted foetus as having postaxial polydactyly. Whole‐exome sequencing identified a missense variant (c.3599C>T, p.A1200V) in exon 20 and a c.834+1G>T variant in exon 7 of *CPLANE1* (NM_023073.3) in the foetus. Sanger sequencing confirmed that these variants came from the parents of the foetus. In this study, we investigated a family with OFDS VI through genetic testing and bioinformatics analysis, which provided powerful help for prenatal diagnosis. Then, we demonstrated that the cell migration rate and the number of cilia were decreased after interference with *CPLANE1* expression in NIH/3T3 cells. After *CPLANE1* knockdown, the Hh signalling pathway was inhibited, and the Hh pathway activator SAG reversed the inhibitory effect. This is the first report of a family with OFDS VI in the Chinese population.

## 1 INTRODUCTION

Oral‐facial‐digital syndrome (OFDS) [OMIM: 277170] is a rare atypical ciliopathy that was first reported in 1941. The affected members exhibited typical craniofacial anomalies and variable occurrence of polydactyly; thus, the syndrome was named OFDS. With the advancement of science and technology, genetic testing and research combined with analyses of the clinical manifestations of patients have revealed that OFDS is a rare genetic disease caused by genetic variants. OFDS mainly manifests with oral and maxillofacial deformities accompanied by hand–foot–skeletal deformities or multiple system deformities, such as kidney and nervous system deformities.^1^ Because malformations of the mouth, face and limbs affect the quality of life of patients and because improper development of the kidneys and nervous system can directly threaten life, the prognosis of OFDS is poor. OFDS is divided into OFDS I‐XIII; OFDS I is inherited in an X chromosome‐dominant manner, while the rest exhibit autosomal recessive inheritance.^2^

OFDS Ⅵ is rarer than OFDS Ⅰ or OFDS Ⅱ and is also known as Varadi syndrome. One study systematically examined 5 unrelated families diagnosed with OFDS VI and summarized the diagnostic features, including molar signs, a tongue hamartoma and/or tongue lobes, an additional frenulum, polydactyly, split toe and/or half toe, and hypothalamic hamartoma.^3^ According to the clinical manifestations of the molar signs on brain magnetic resonance imaging (MRI), OFDS VI is classified as a disease related to Joubert syndrome.^4^

The current evidence suggests that the only gene mutated in OFDS Ⅵ is *CPLANE1* (ciliogenesis and planar polarity, also known as C5orf42/Jbts17).^5^ The *CPLANE1* gene is located on chromosome 5p13.2. It contains 52 exon regions and encodes a protein of 3198 amino acids, CPLANE. CPLANE may be involved in the transport function of cilia or signal transduction pathways related to cilia.

In this study, a 21^+3^‐week‐old Chinese pregnant woman (gravida 3, para 0) was first diagnosed at the Prenatal Diagnosis Center of Guizhou Medical University Affiliated Hospital, Guiyang, China. It was her 3rd pregnancy, and the foetus was the proband in this study. We performed an ultrasound examination, and the results showed that the foetus had a Blake cyst in the posterior fovea and foot polydactyly. Therefore, the patient decided to terminate the pregnancy and conduct further genetic molecular analysis. After induction of labour, we found that the feet of the foetus had six toes. Upon questioning, the woman reported that her husband was 27 years old and in good health. Both the husband and wife denied marriage of close relatives and denied a family history of illness. Karyotype analysis and copy number variation (CNV) analysis revealed no chromosomal abnormalities in the foetus. Genomic DNA was extracted from the umbilical cord of the foetus. Through whole‐exome sequencing, a compound heterozygous variant was identified in exon 20 of the *CPLANE1* gene of the foetus (splice variant: c.834+1G>T, combined missense variant: c.3599C>T, p.A1200V). Sanger sequencing confirmed that both parents of the foetus were carriers of the variant. Our current research has identified the first case of OFDS VI in the Chinese population. Based on the results of the molecular diagnosis, with our help, the family has now given birth to a healthy boy. We emphasize the significance of whole‐exome sequencing for the diagnosis of ciliary diseases.

## 2 MATERIALS AND METHODS

### 2.1 Patients and families

A family with OFDS VI was selected from the Prenatal Diagnosis Center of the Affiliated Hospital of Guizhou Medical University, Guiyang, China. We collected the peripheral blood of adults in the family, the umbilical cord of the foetus and amniotic fluid for experiments. This study was approved by the Ethics Committee of the Affiliated Hospital of Guizhou Medical University (approval 2019: no. 171; Guiyang, China). We obtained written informed consent from the participants in this study.

### 2.2 Karyotype and chromosomal microarray analyses

To analyse the structures of all the chromosomes in the foetus, we performed standard G‐banding karyotyping. Next, to confirm the presence of CNVs in the foetus, chromosome microarray analysis was performed.

### 2.3 Whole‐exome sequencing and identification of variants

We extracted foetal genomic DNA from the umbilical cord and amniotic fluid of adults and foetuses. The DNA extraction process was carried out in strict accordance with the kit instructions (Zeesan, China). The foetal genomic DNA was sent to Berry Genomics Biotechnology Co., Ltd., for whole‐exome sequencing and Sanger sequencing (NextSeq 2000, Illumina, USA). By consulting the NCBI, OMIM and other related databases and using the Verita Trekker variant site detection system and the Enliven^®^ variant site annotation interpretation system of Berry Genomics, we annotated the identified variants. Finally, the annotated variants were verified by Sanger sequencing.

### 2.4 Interference with *CPLANE1* expression in NIH/3T3 cells

According to the *CPLANE1* gene sequence in mice (Gene ID: 73692), we designed and synthesized an siRNA oligonucleotide sequence that interferes with the expression of the mouse gene (Jima, China). The siRNA was transfected into NIH/3T3 cells (from the National Collection of Authenticated Cell Cultures, ATCC: CRL‐1658) with Lipofectamine 2000 (Thermo, USA) using the following primers: F: 5′‐GCAUACUUCUGUGGGAAUATT‐3′ and R: 5′‐UAUUCCCACAGAAGUAUGCTT‐3′. The siRNA with the highest transfection efficiency was obtained by RT–qPCR screening.

### 2.5 Immunofluorescence study of NIH/3T3 cells

The bottom of a confocal dish was coated with 1 mg/ml poly‐D‐lysine (Sigma, USA). The transfected NIH/3T3 cells (1 × 10^5^) were cultured in the confocal dish. After adding 1 ml of 4% paraformaldehyde (Solarbio, China) to fix the cells, the cells were washed with PBS (Sigma, USA) 3 times. After adding 0.5% Triton X‐100 (Beyotime, China) for permeabilization, the cells were washed in PBS 3 times. After the cells were incubated with 10% goat blood serum (Boster, China) for 2 h at room temperature, a diluted primary antibody was added to the centre of the dish, and the small dish was incubated overnight in a humidified box at 4°C. After rewarming at room temperature for 1 h, the cells were soaked in PBS and washed 3 times. A diluted fluorescent secondary antibody was added to the centre of the dish, and the cells were incubated for 1 h at room temperature in the dark before being washed with PBS 3 times. DAPI staining solution (300 µl, Solarbio, China) was added to the centre of the dish, and the cells were incubated for 5 min in the dark before being washed 3 times with PBS. Anti‐fluorescence quencher (200 µl, Solarbio, China) was added to the centre of the dish. An LSM700 laser confocal microscope (ZEISS, Germany) was used to observe and image the cells. Immunofluorescence analysis was performed with the following antibodies: anti‐acetylated‐α‐Tubulin (K40) (1:500, Abcam, USA, ab24610), anti‐γ‐Tubulin (1:100, Proteintech, China, Cat No. 15176‐1‐AP), an Alexa Fluor 488 antibody (1:500, CST, UK, #4416) and an Alexa Fluor 594 antibody (1:500, CST, UK, #8890). All experiments were carried out independently at least three times.

### 2.6 Transwell study on NIH/3T3 cells

After transfection for 24 h, NIH/3T3 cells were digested with 0.125% trypsin (Gibco, USA) and then centrifuged (800 rpm, 5 min), and the supernatant was collected. The cells were resuspended in 2 ml of DMEM (Gibco, USA) and counted. Cells (2 × 10^4^/well) were added to a transwell chamber. Next, 10% serum‐free DMEM (Gibco, USA) was added to a 24‐well plate, and the transwell chamber (Corning, USA) was placed into the 24‐well plate. The cells were incubated for 24 h in an incubator. After 24 h, the chamber was removed and rinsed gently with PBS 3 times, and the cells on the bottom of the chamber were gently wiped off with a sterile cotton swab. Paraformaldehyde (4%, Solarbio, China) was added to the blank well and the small chamber, and the cells were fixed for 15 min. The paraformaldehyde was removed, and the plate was rinsed with PBS twice. The chamber was then air‐dried upside down. Crystal violet (0.1%, Solarbio, China) was added to the blank well and the chamber and allowed to stain the cells for 15 min. Then, the crystal violet was removed, and the chamber was rinsed with PBS 3 times and air‐dried upside down. Images were obtained, and the cells were counted under a microscope (Nikon, Japan). All experiments were carried out independently at least 3 times.

### 2.7 Construction of a *CPLANE1*: c.3572 C>T variant mouse model

CRISPR Cas9 technology was used to construct a *CPLANE1*: c.3572 C>T variant mouse model. A 5′ homology arm‐*CPLANE1* variant donor‐3′ homology arm homologous recombinant vector was constructed. The recombinant vector, Cas9 mRNA and sgRNA were injected into the fertilized eggs of mice. The two‐cell embryos were transplanted into the oviducts of surrogate mother mice, and the *CPLANE1* gene variant site was finally inserted. The c.3572 C > T variant mouse model was obtained from Shanghai Paizhi Biological Technology Co., Ltd. (Shanghai, China).

The designed gRNAs were as follows:

sgRNA‐1: GCGAGTTCTCCTGCTTTTCCGGG (PAM + target sequence).

sgRNA‐2: TCCTGCTTTTCCGGGCATCTCGG (PAM + target sequence).

sgRNA‐3: GAAAAGAACACCGAGATGCCCGG (PAM + target sequence).

### 2.8 Western blot analysis

RIPA lysis buffer (plus PMSF) was used to lyse the cells to extract the total proteins, which were separated by polyacrylamide gel electrophoresis and transferred to PVDF membranes. After membrane blocking, the appropriate primary antibodies (1:1000, rabbit anti‐mouse, anti‐shh, CST, USA, anti‐Ptch1/Gli1, R&D, USA, anti‐Smo/β‐actin, Servicebio, China, and anti‐CPLANE1, ABclonal, China) were added to the membrane, which was incubated overnight at 4°C. Next, the secondary antibody (1:20000, goat anti‐rabbit antibody, Bioworld, China) was added to the membrane, which was incubated at room temperature for 1 h. Finally, the bands were imaged using a gel imaging system.

### 2.9 Indirect immunofluorescence staining to detect Gli1 entering the nucleus

Cells were immersed in PBS and seeded in glass slides at a concentration of 1 × 10^4^/ml. The slides were fixed in 4% paraformaldehyde for 15 min, immersed in PBS 3 times for 3 min each time and subsequently immersed in 0.5% Triton X‐100 in PBS at room temperature for 20 min. Next, the cells were blocked with 5% skim milk powder in PBST for 30 min at room temperature, and the following diluted primary antibodies were added: rabbit anti‐mouse and anti‐Gli1 (R&D, USA). The antibodies used were rabbit anti‐human and were diluted at a concentration of 1:200. The cells were incubated overnight at 4°C and subsequently incubated with a fluorescent secondary antibody (1:500, goat anti‐rabbit antibody, CST, USA) for 1 h at room temperature. An appropriate amount of DAPI (1:5000, Thermo, USA) was added. The cells were incubated at room temperature for 5 min in the dark, and the nucleation status of Gli1 in each treatment group was observed under an inverted fluorescence microscope (Nikon, Japan).

### 2.10 Rescue experiment with the activator SAG

siRNA was transfected into NIH/3T3 cells. Twenty‐four hours after transfection, 40 μL of 100 μmol/L SAG (final concentration: 4 μmol/L, HY‐12848, MCE, USA) was added to the 6‐well plate, and the plate was incubated for 48 h. RNA and protein were extracted separately for qRT–PCR and Western blot detection.

## 3 RESULTS

### 3.1 Human subjects

**FIGURE 1:**
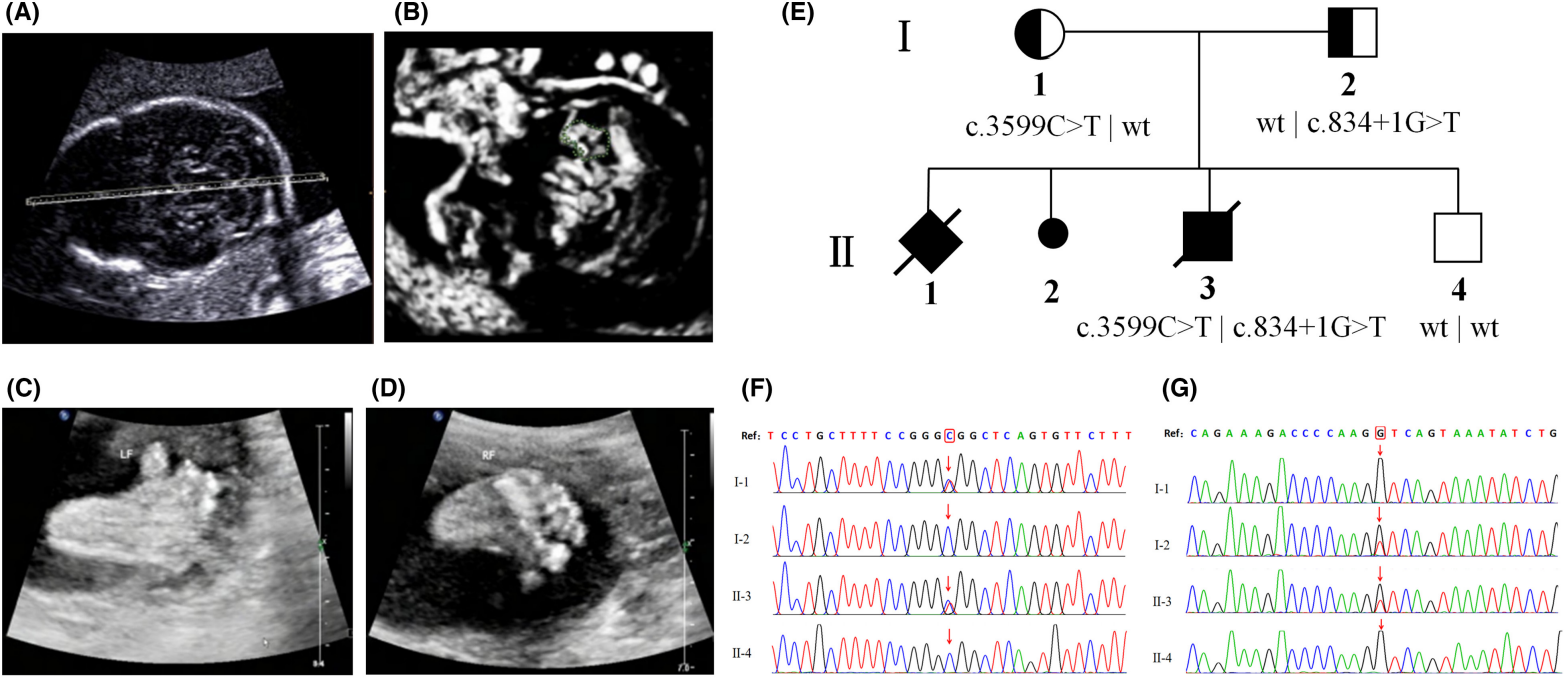
Pedigree of the Chinese family with OFDS Ⅵ. Ultrasonography examination revealed that proband's posterior fossa “Blake” cyst (A‐B). Ultrasonography examination revealed that proband's two feet with six‐toed deformity (C‐D). Pedigree of the described nonconsanguineous Chinese family with OFDS Ⅵ (E). Squares and circles denoted males and females respectively. Individuals labelled with a solidus were deceased. Roman numerals indicate generations. Sanger sequencing in the *CPLANE1* of the family (F‐G). The reference sequence NM_023073.3 of *CPLANE1* gene was used. I‐1: proband's mother, I‐2: proband's father, II‐3:proband, II‐3:proband's brother. *CPLANE1*: c.3599C>T, p.A1200V, proband and his mother are carriers, his father and brother are wild type (F). *CPLANE1*: c.834+1G>T, proband and his father are carriers, his mother and brother are wild type (G)

In the present study, we investigated a 26‐year‐old pregnant Chinese woman who had a history of adverse pregnancy outcomes. This Chinese family was confirmed to be nonconsanguineous. In 2015, the woman became pregnant for the first time. Ultrasonography at 21^+3^ weeks of pregnancy showed foetal corpus callosum hypoplasia, cerebellar vermis hypoplasia and polydactyly after the biaxial axis. The pregnancy was terminated. The sex of the foetus was unknown. The woman's second pregnancy in 2016 was spontaneously aborted when the foetus was approximately 3 months old, and the woman did not undergo relevant prenatal checkups. In 2017, the woman came to our hospital for a third pregnancy. When she was 23^+1^weeks pregnant, we found through ultrasound examination that the foetus had a posterior fossa Blake cyst and polydactyly (Figure 1A‐D). Therefore, the woman decided to terminate the pregnancy and conduct further genetic molecular analysis. She had her fourth pregnancy in 2019 and gave birth to a healthy male baby in 2020 (Figure 1E).

**FIGURE 2:**
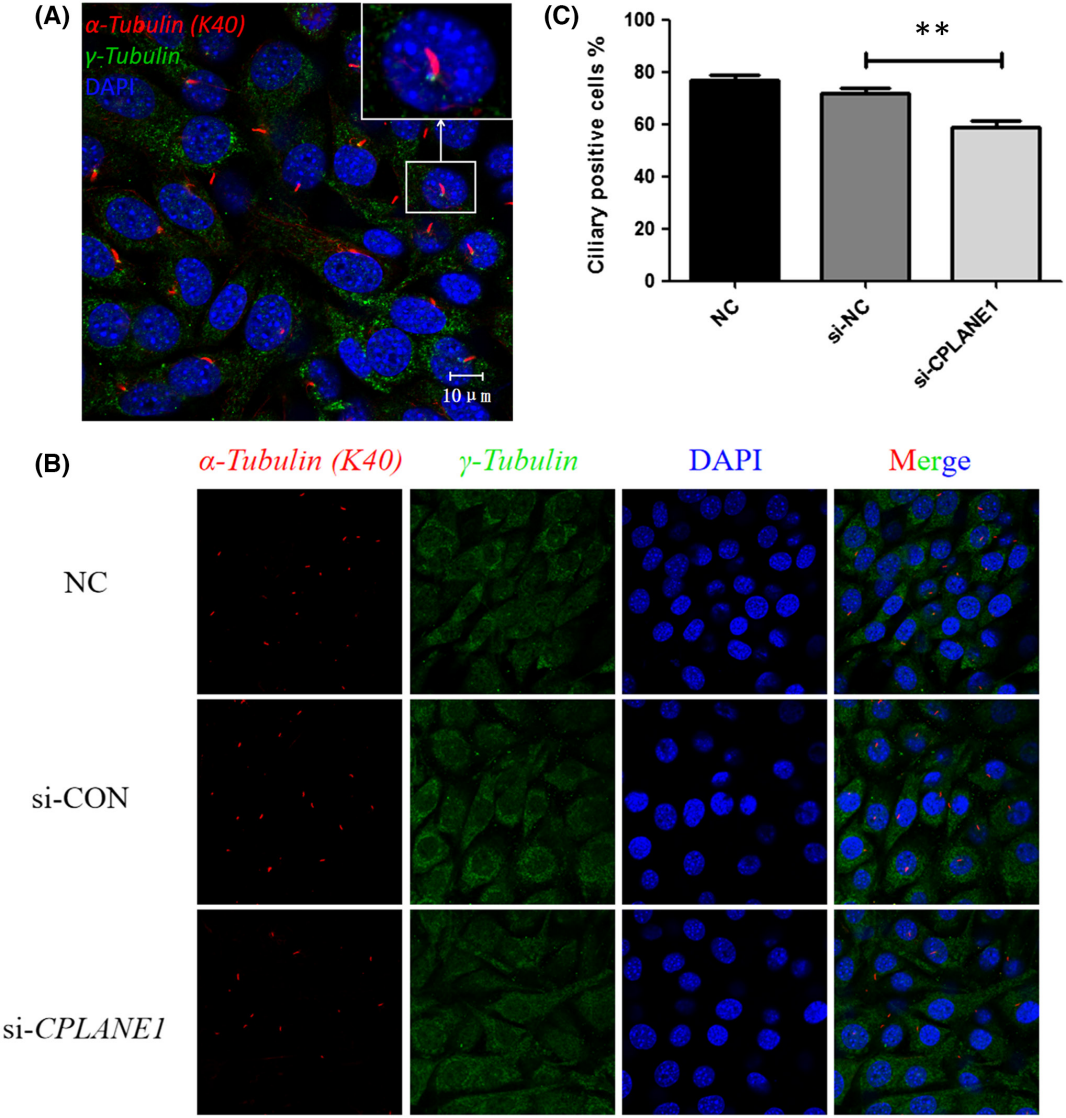
Localization of cilia in NIH/3T3 cells and count the number (630×). DAPI, for nuclear staining (blue); ciliary body, anti‐α‐tubulin (K40) followed by an Alexa Fluor 594‐conjugated secondary antibody (red); ciliary base, γ‐Tubulin antibody followed by an Alexa Fluor 488‐conjugated secondary antibody (green); Merge, DAPI nuclear staining plus ciliary staining. (A) Localization of cilia in NIH/3T3 cells. (B) Count the number of cilia in NIH/3T3 cells. (C) Bar graph shows that the ciliary positive is lower in si‐CPLANE1 cells (58.61%) than those in si‐CON cells (71.67%). **p < 0.01

### 3.2 Karyotype and chromosomal microarray analyses

CNV sequencing showed that the pregnant woman, the proband (II‐3) and the proband's brother (II‐4) did not exhibit chromosomal aneuploidy or known genome copy number variants of more than 100 kb. The g‐band chromosome karyotype analysis results were normal.

### 3.3 Whole‐exome sequencing and Sanger sequencing identified a compound heterozygous variant in *CPLANE1*

According to the whole‐exome sequencing and Sanger sequencing results, we found that the pregnant woman was a carrier of the *CPLANE1*: c.3599C>T p.A1200V missense variant and that her husband was a carrier of the *CPLANE1*: c.834+1G>T variant. The proband carried the two variants at the same time, representing a compound heterozygous mutant. The proband's brother (II‐4) did not carry the above two variants. The variant of the pregnant woman was inherited from her mother, and the variant of her husband was inherited from his mother (Picture 1 F‐G).

### 3.4 The number of cell cilia decreases after interference with *CPLANE1* expression

After the cells were transfected for 48 h, the cells were stained for immunofluorescence analysis. The cilia body was marked by acetylated‐α‐Tubulin (K40) and showed red fluorescence. The cilia base was marked by γ‐Tubulin and showed green fluorescence. The nucleus was marked by DAPI and showed blue fluorescence. Under the oil objective of a laser confocal microscope, each cell had only one cilium, the length of which was typically between 5 and 10 µm.

The cells were photographed, the cilia were counted under the laser confocal microscope, and the numbers of positive cilia were compared among the groups. The results showed that si‐*CPLANE1*‐transfected cells had significantly fewer cilia than control siRNA (si‐CON)‐transfected cells (*p* < 0.01) (Figure 2).

### 3.5 The cell migration rate decreases after interference with *CPLANE1* expression

**FIGURE 3:**
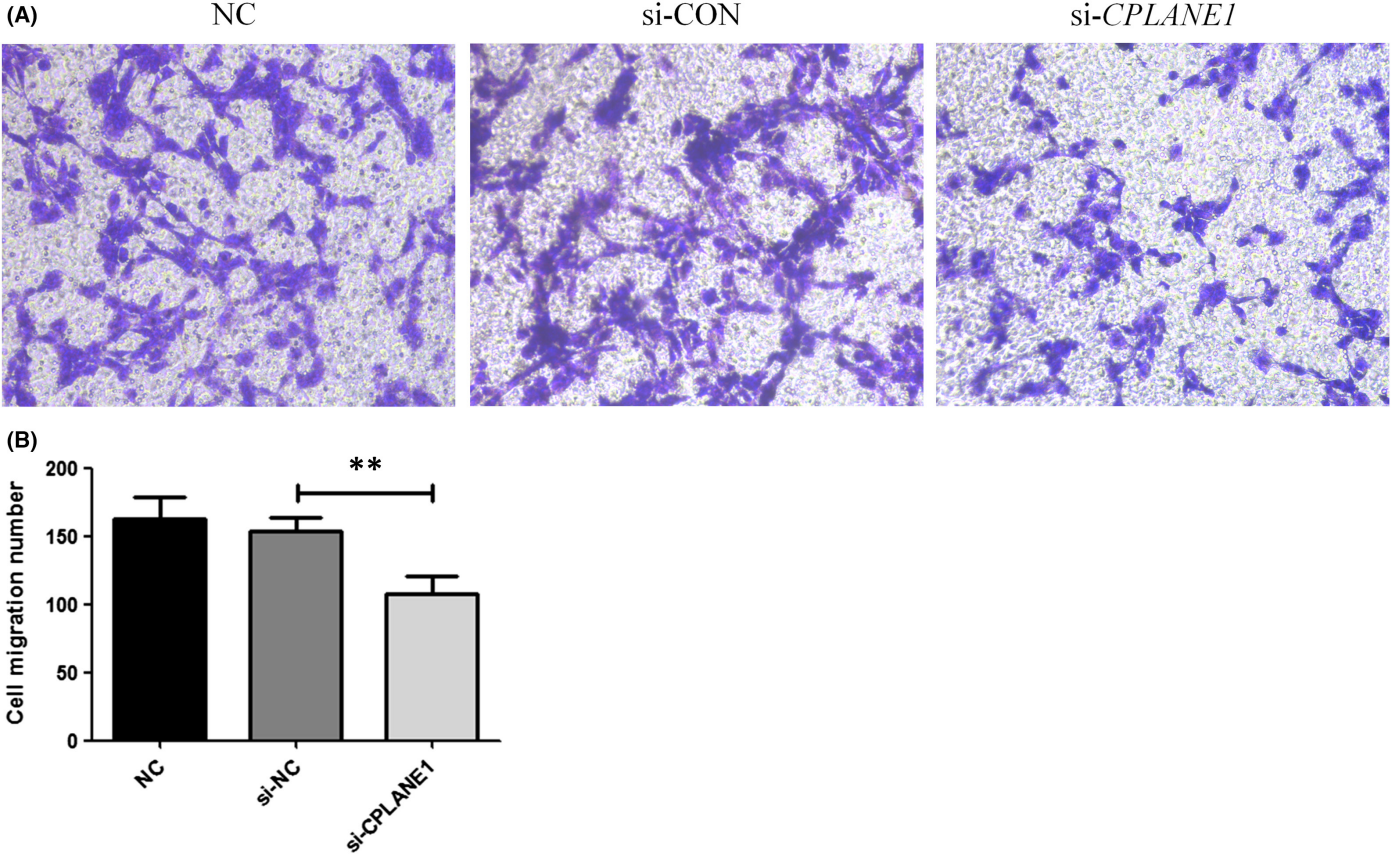
Cell migration analysis. (A) Cell migration analysis by transwell in NIH3T3 cells for 24 h after seeding. (B) Bar graph shows that the ciliary positive is lower in si‐CPLANE1 cells (*n* = 108 cells) than those in si‐CON cells (n = 153 cells). **p < 0.01

After the cells were transfected for 24 h, they were digested with trypsin, recounted, plated in a transwell chamber and cultured for another 24 h. The cell penetration was observed 48 h after transfection. The results showed that significantly fewer cells passed through the membrane in the si‐*CPLANE1* group than in the si‐CON group (*p* < 0.01) (Figure 3).

### 3.6 A *CPLANE1* variant affects the development of fertilized eggs

In our previous study, we found a family with suspected OFDS VI through prenatal ultrasound examination and whole‐exome sequencing in the early stage. The pregnant women in this family had a history of difficult pregnancies and childbirth. In the third pregnancy of the woman in that study, ultrasonography revealed foetal malformations. Through whole‐exome sequencing, a missense variant of *CPLANE1* was discovered.

**FIGURE 4:**
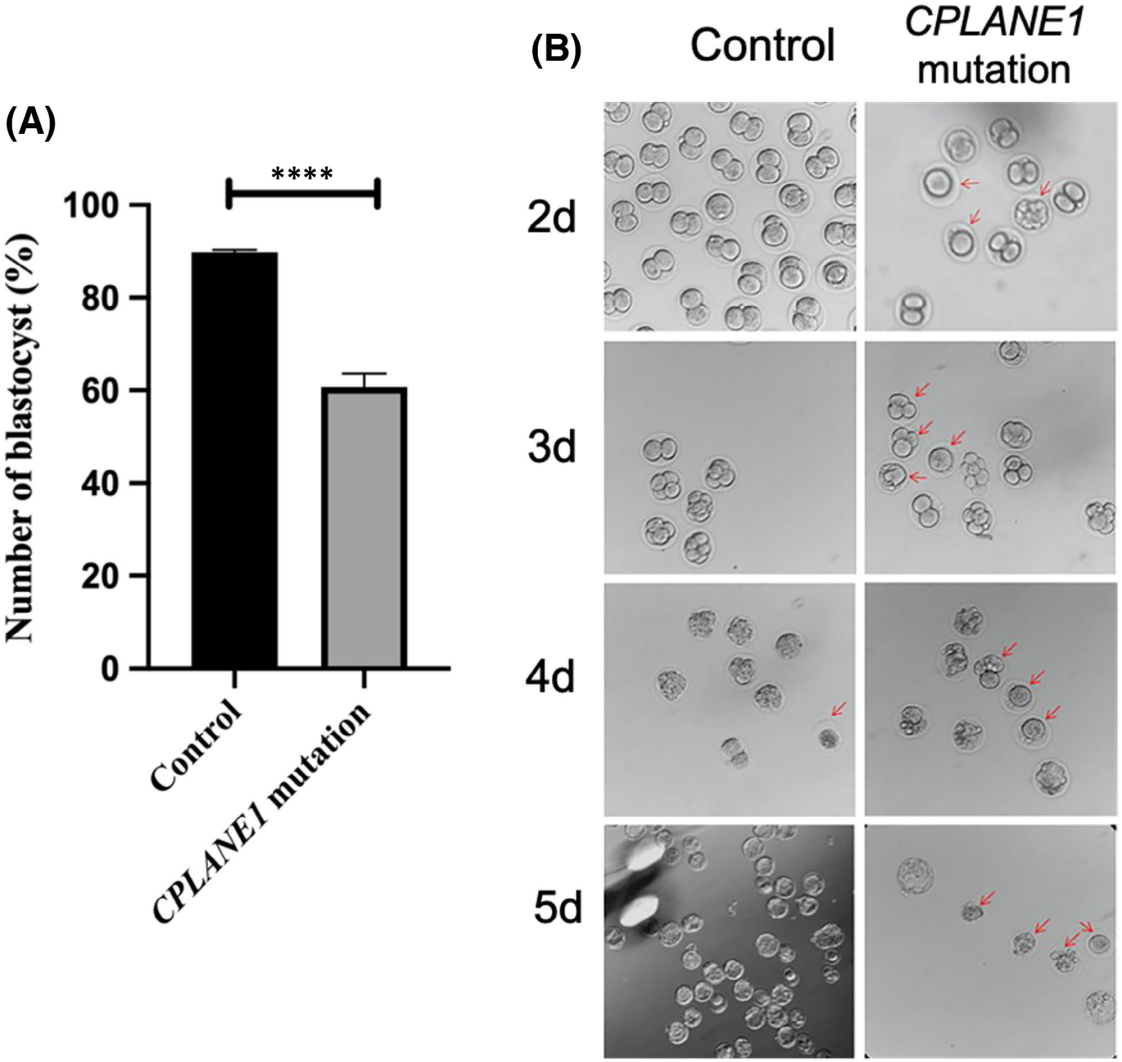
*CPLANE1* mutations affect the development of fertilized eggs. (A) Statistics of the development rate of blastocysts in fertilized eggs on the 2 days after microinjection. (B) Observe the changes of fertilized eggs at different time points after microinjection. The red arrow indicates a fertilized egg with abnormal structure

To study whether the *CPLANE1* variant could cause foetal malformations, we used CRISPR–Cas9 technology to construct a mouse model of the *CPLANE1* gene missense variant, designed an sgRNA targeting the 21st exon of CPLANE1 and constructed a 5′ homology arm‐*CPLANE1* variant donor‐3′ homology arm homologous recombination vector. We microinjected Cas9 mRNA, the sgRNA and the donor template into fertilized mouse eggs. The fertilized eggs developed abnormally after *CPLANE1* editing. On the 2nd day after microinjection, we selected the fertilized eggs that had developed to the two‐cell stage and continued to observe them. We simultaneously quantified the blastocyst development rate of the fertilized eggs. Compared with that in the control group (without sgRNA), the percentage of fertilized eggs that developed into blastocysts in the *CPLANE1* variant group was significantly lower. The rate in the control group was approximately 90%, and the rate in the *CPLANE1* variant group was approximately 63% (*p* < *0*.*0001*) (Figure 4A). By the 5th day, the embryos in the control group had developed into blastocysts. In contrast, in the *CPLANE1* variant group, fertilized eggs developed to the two‐cell stage, after which the development of some of the embryos stagnated. Apoptosis began to appear around the 4th day, causing the embryos to fail to develop normally (Figure 4B). The above results confirm that the *CPLANE1* variant affects the normal development of embryos.

### 3.7 The Hh signalling pathway is inhibited in *CPLANE1*‐knockdown NIH/3T3 cells

**FIGURE 5:**
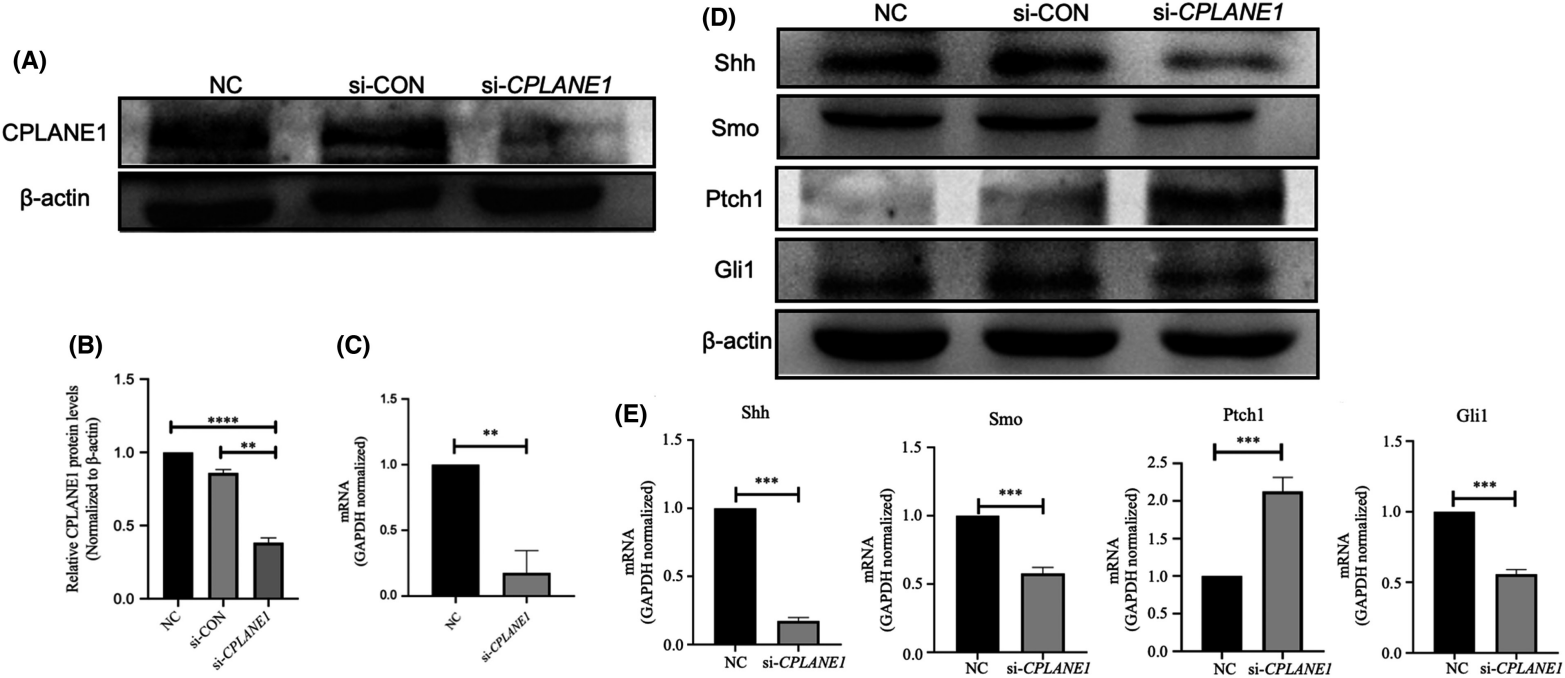
The Hh signal pathway was inhibited in CPLANE1 knock down NIH/3T3 cells. Transfection si‐CPLANE1/si‐CON into NIH/3T3 cells, at 48 h post transfection, the expression of CPLANE1 detected by Western blot (A‐B) and qRT‐PCR (C). The expression of Shh, Smo, Ptch1 and Gli1 was detected by Western blot (D) and qRT‐PCR (E). NC and si‐CON were used as negative control. Data are shown as the mean ± SEM. **p < 0.01, *** *p* < 0.001, ****p < 0.0001. Representative results of 3 independent experiments are shown

si‐*CPLANE1* was transiently transfected into NIH/3T3 cells, and cells were collected 48 h after transfection. Western blotting and qRT–PCR were used to detect the efficacy of *CPLANE1* silencing. The results showed that, compared with the NC group and the si‐CON group, the expression level of *CPLANE1* was significantly reduced in *CPLANE1* knockdown NIH/3T3 cells (Figure 5A‐C).

To study whether *CPLANE1* is involved in the regulation of the Hh signalling pathway, we transiently transfected si‐*CPLANE1* into NIH/3T3 cells and collected the cells 48 h after transfection. Western blot and qRT–PCR were used to detect the expression of Shh, Smo, Ptch1 and Gli1. The results showed that compared with the negative control (NC) and si‐CON groups, the *CPLANE1*‐knockdown group had significantly lower expression levels of Shh, Smo and Gli1 *(p* < *0*.*05)* and a significantly higher expression level of Ptch1 *(p* < *0*.*01)* (Figure 5D‐E).

We transiently transfected si‐*CPLANE1* into NIH/3T3 cells and used indirect immunofluorescence staining to detect the expression of the transcription factor Gli1 and the nucleus. The results showed that the NC and si‐CON groups had obvious green fluorescent signals, and the green fluorescence was distributed on the nucleus. In the si‐*CPLANE1* group, the green fluorescent signal was weak, and no green fluorescence was distributed on the nucleus (Supplementary Figure S1).

The above results revealed that knocking down *CPLANE1* can inhibit the expression of Ptch1, inhibit the binding of Shh and Smo, and reduce the expression of Gli1 and the ability of Gli1 to enter the nucleus, thereby inhibiting signalling through the Hh signalling pathway. The above results confirm that *CPLANE1* is involved in promoting signal transmission through the Hh signalling pathway.

### 3.8 The Hh pathway activator SAG can reverse the inhibition of the Hh signalling pathway in *CPLANE1*‐knockdown NIH/3T3 cells

**FIGURE 6:**
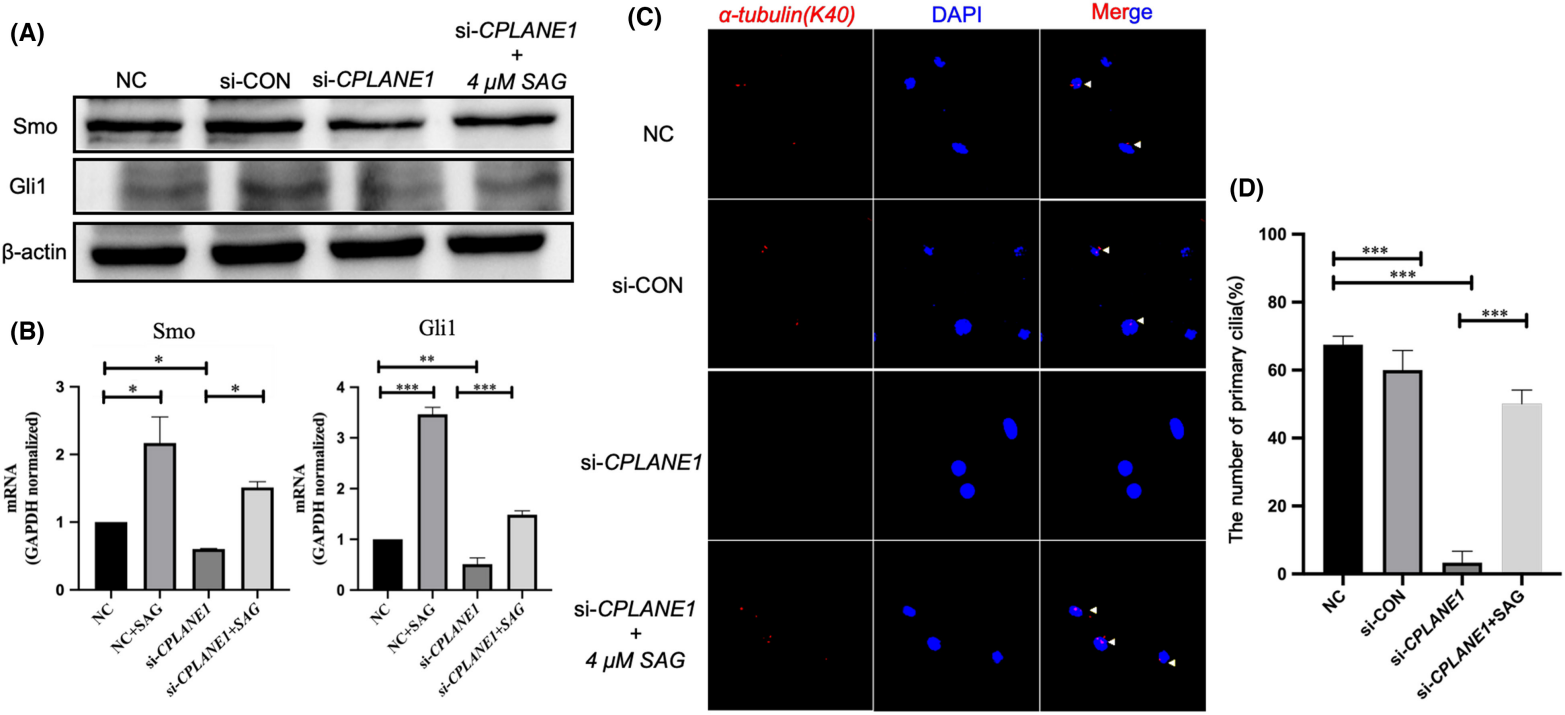
The Hh pathway activator SAG can reverse the inhibitory effect of Hh signal pathway in CPLANE1‐kncokdown NIH/3T3 cells. Transfection si‐CPLANE1/si‐CON into NIH/3T3 cells, at 24 h post transfection, added 4 μM SAG into si‐CPLANE1 and incubated for 24 h. Analysis of Smo and Gli1 by Western blot analysis (A) and qRT‐PCR analysis (B) at 48 h post exposed. (C) The number of primary cilia was taken by laser scanning confocal microscopy, anti‐α‐tubulin(K40) (Red) to visualize the primary cilia and with DAPI (Blue) to visualize the nuclei. (D) Statistical analyses of the number of primary cilia in each group. NC and si‐CON were used as negative control. Data are shown as the mean ± SEM. *p < 0.05. Representative results of 3 independent experiments are shown

To observe whether the Hh pathway activator SAG could reverse the inhibitory effect of *CPLANE1* knockdown on the Hh signalling pathway, we transfected si‐*CPLANE1* into NIH/3T3 cells and added 4 μM SAG. The expression of Smo and Gli1 was analysed using Western blotting and qRT–PCR. The results showed that the expression levels of Smo and Gli1 were significantly higher in the group of SAG‐treated *CPLANE1*‐knockdown cells than in the si‐*CPLANE1* group *(p* < *0*.*05)* (Figure 6A,B, Supporting Information Figure S2). The above results confirm that the Hh pathway activator SAG can reverse the inhibitory effect of *CPLANE1* knockdown in cells.

We next detected primary ciliated cells by laser confocal microscopy. The results showed that the NC group and si‐CON group had obvious red fluorescent signals, while red fluorescence was not detected in the si‐*CPLANE1* group. When SAG was added to *CPLANE1*‐knockdown cells, obvious red fluorescence was detected (Figure 6C). We quantified the primary cilia in each group and found more primary cilia in the SAG‐treated *CPLANE1*‐knockdown group than in the si‐*CPLANE1* group *(p* < *0*.*001)* (Figure 6D). The above results confirm that adding SAG can reverse the effect of *CPLANE1* knockdown on the number of primary cilia.

## 4 DISCUSSION

With the improvement of foetal ultrasound accuracy and the development of second‐generation sequencing technology, prenatal diagnosis of OFDS has become possible. We found a family with OFDS VI through foetal ultrasound examination and whole‐exome sequencing. In this family, the parents of the proband carried the *CPLANE1*: c.3599C>T variant and the c.834+1G>T variant, and both passed them to the proband. We conducted a database query and bioinformatics analysis on these two variants. The variant frequencies of the 3599C>T variant in the 1000G, ExAC and gnomAD databases were 0, 0 and 5.798e^−05^. The CADD and GERP scores were conservatively predicted, and the results showed that the site is evolutionarily conserved and has potential functional impact. The protein function was predicted with SIFT, Polyphen and other tools. MutationTaster predicted that c.3599C>T is a pathogenic variant. The c.834+1G>T variant occurs in the splicing region, which leads to changes in protein function. This variant was not found in the 1000G, ExAC and gnomAD databases. The CADD and GERP scores were conservatively predicted, and the results showed that the site has evolved. The variant is conservative and has potential functional impact. MutationTaster predicted the c.834+1G>T variant to be a pathogenic variant. Studies have shown that the c.3599C>T variant in OFDS VI patients is concentrated within amino acids 1127–1345, and this region may be functionally related.^6^ In our study, the c.3599C>T (p.A1200V) variant was located in the above interval, the proband's feet exhibited preaxial polydactyly, and the hands of the first foetus exhibited postaxial polydactyly. These findings supported the diagnosis of OFDS VI. According to the American Academy of Medical Genetics and Genomics (ACMG) variant guidelines,^7^ the c.3599C>T variant can be classified as likely pathogenic (LP) with 2 moderate (PM2, PM3) and 2 supporting (PP3, PP4) bodies of evidence, and the c.834+1G>T variant can be classified as pathogenic (P) with 1 very strong (PVS1) body of evidence, 1 moderate (PM2) body of evidence and 1 supporting (PP4) body of evidence.

Existing studies suggest that cilia often play important roles in the development and proliferation of stem cells, organs and germ cells. Cilia structural dysfunction and abnormal Hedgehog signalling pathways can lead to ciliary diseases.^8^ In this experiment, we conducted a preliminary study on the *CPLANE1* gene in NIH/3T3 cells, laying the foundation for subsequent studies on the mechanism of ciliary disease. In this experiment, *CPLANE1*‐siRNA was transfected into NIH/3T3 cells in a liposome‐mediated manner to establish a *CPLANE1*‐knockdown embryonic fibroblast model. A transwell migration test revealed that the cell migration rate after *CPLANE1* knockdown was significantly lower than that of the NC group (*p* < 0.01). Immunofluorescence staining of the cilia body and base was used to observe and count the cilia in the cells before and after transfection. The percentage of positive cilia in each control group ranged from 70% to 75%, and there were no significant differences. The number of positive cilia in the CPLANE1‐siRNA transfection group was approximately 57%, which was significantly lower than the percentage in the NC group (*p* < 0.01). The cilia in each group were typically between 3 and 10 µm long; most of the cells exhibited visible intact ciliated bodies and basal parts, but some of the basal parts of cilia were not visible.

According to the existing literature, *CPLANE1* gene knockout can cause abnormal embryonic development, but there have been no reports on models with *CPLANE1* gene missense variants. *CPLANE1* gene missense variants have failed to be constructed using CRISPR–Cas9 technology because the *CPLANE1* gene A variant is a pathogenic variant that can cause developmental abnormalities and because the off‐target effects of CRISPR–Cas9 technology lead to embryonic death.

Primary cilia can be regarded as cell ‘antennas’ of sorts. In addition to acting as sensors, primary cilia play a role in the transmission of mechanical and chemical stimuli. Furthermore, primary cilia play an important role in regulating cell signal transduction through pathways such as the Hh signalling pathway. Primary ciliogenesis starts from the formation of the mother's central cell template. Under the regulation of a variety of proteins, the assembly of cilia is regulated by vesicular transport, BBS complex interactions and intraflagellar transport proteins.^9^, ^10^, ^11^ The regulation of protein transfer in and out of primary cilia is mediated by a special structure of the primary cilia: the transition zone.^12^, ^13^, ^14^ Primary cilia abnormalities lead to the development of other abnormalities and metabolic diseases, such as kidney cysts and abnormal brain development.^15^, ^16^, ^17^ Variants or deletions of *CPLANE1* are closely related to a variety of defects, among which the *CPLANE1* variant is a common cause of Joubert syndrome. The mouse embryonic fibroblast cell line NIH/3T3 has primary cilia in the G2/S phase.^18^ In the past, it was thought that *CPLANE1* interacted with the NPHP1 gene in the primary ciliary transition region to cause the occurrence of Joubert syndrome.^11^ To confirm whether *CPLANE1* affects the number of primary cilia, we used the primary ciliary body marker acetylated‐α‐Tubulin (K40) and performed indirect immunofluorescence staining to label the primary cilia. The laser confocal microscopy results showed that *CPLANE1* was not detected after silencing. The red fluorescent signals indicated that silencing *CPLANE1* significantly reduced the number of primary cilia, confirming that *CPLANE1* plays an important role in maintaining the number of primary cilia. Studies have shown that diseases caused by an abnormal number of primary cilia are often caused by variants in related genes in the transition region.^12^ Currently, there are reports in the literature that *CPLANE1* may itself encode a protein of the primary ciliary transition region. In follow‐up research, we will analyse the location of *CPLANE1* in primary cilia and lay a theoretical foundation for further research on the influence of *CPLANE1* on the number of primary cilia.

Recent studies have found that *CPLANE1* is located in the transition zone of primary cilia. Studies have also shown that related gene variants in the primary ciliary transition zone can affect the function of the Hh signalling pathway, leading to the occurrence of diseases.^19^ The *CPLANE1* variant causes an abnormal number of primary cilia, which affects the Hh signalling pathway transduced by the primary cilia, leading to the occurrence of Joubert syndrome.^12^ To verify whether *CPLANE1* affects the number of primary cilia by regulating the Hh signalling pathway, we silenced *CPLANE1* and detected the expression levels of Hh pathway components. We found that the expression of Shh and Smo was downregulated, the expression of Ptch1 was upregulated, and the expression of the transcription factor Gli1 was downregulated. The ability of Gli1 to enter the nucleus was weakened, which inhibited the function of the Hh signalling pathway. When the Hh signalling pathway activator SAG was added to NIH/3T3 cells with *CPLANE1* silencing, the expression levels of Smo and Gli1 increased significantly, as determined by indirect immunofluorescence staining. The results also confirmed that the number of primary cilia was significantly reduced after *CPLANE1* was silenced but that addition of the activator SAG restored the number to normal. The above results confirm that *CPLANE1* plays an important role in maintaining the number of primary cilia.

## CONFLICT OF INTEREST

The authors confirm that there are no conflicts of interest.

## AUTHOR CONTRIBUTION

**Wen Qian:** Data curation (equal); Formal analysis (equal); Investigation (equal); Methodology (equal); Validation (equal); Visualization (equal); Writing – original draft (equal); Writing – review & editing (equal). **Xinlei Liu:** Data curation (equal); Methodology (equal); Supervision (equal); Validation (equal); Visualization (equal); Writing – original draft (equal); Writing – review & editing (equal). **Zhengrong Wang:** Conceptualization (equal); Data curation (equal); Investigation (equal); Methodology (equal); Project administration (equal); Software (equal). **Yongjie Xu:** Supervision (equal); Visualization (equal); Writing – original draft (equal); Writing – review & editing (equal). **Jingzhi Zhang:** Investigation (equal); Resources (equal). **Qiang Zhong:** Investigation (equal); Methodology (equal); Resources (equal); Software (equal). **Cheng cheng Li:** Investigation (equal); Resources (equal); Software (equal). **zunlun zhou:** Conceptualization (equal); Project administration (equal); Resources (equal); Writing – review & editing (equal). **Wei Pan:** Conceptualization (lead); Data curation (lead); Funding acquisition (lead); Methodology (lead); Project administration (lead); Resources (lead); Validation (equal); Writing – review & editing (lead). **Haizhi Li:** Data curation (equal); Investigation (equal); Writing – original draft (equal); Writing – review & editing (equal). **Liying Zhu:** Methodology (equal); Supervision (equal); Writing – review & editing (equal).

## Supporting information

Fig S1

Fig S2

## Data Availability

The datasets used and/or analysed during the current study are available from the corresponding author on reasonable request.
