# Supplementary figures and images for: Whole‐exome sequencing identified novel variants in CPLANE1 that causes oral‐facial‐digital syndrome Ⅵ by inducing primary cilia abnormality
Source: J Cell Mol Med. 2022 May 18;26(11):3213–22. doi: 10.1111/jcmm.17326 (PMC9170817; doi:10.1111/jcmm.17326)

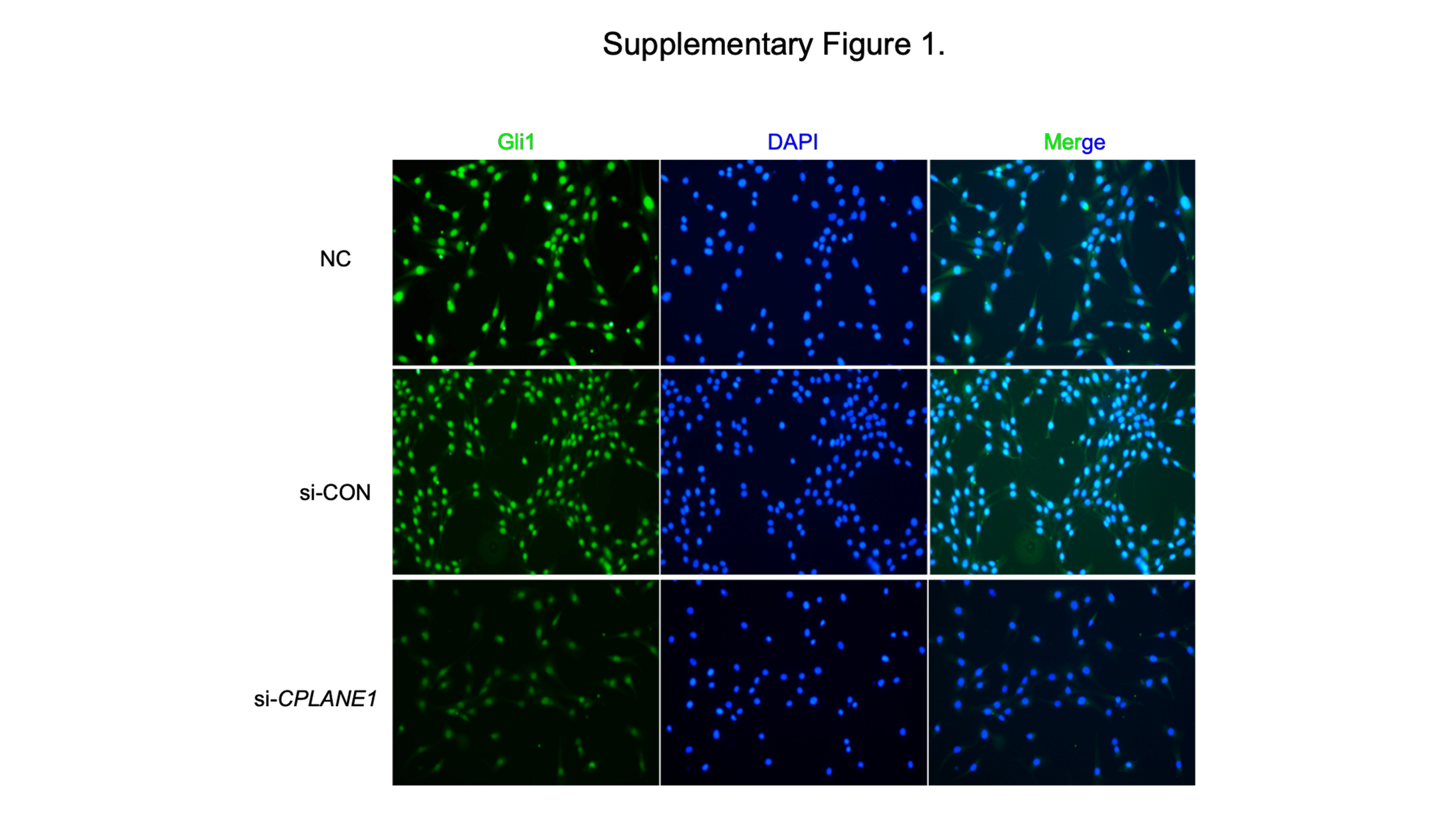

Supplement: Supplementary file 1 — Fig S1 [file JCMM-26-3213-s002.tif]

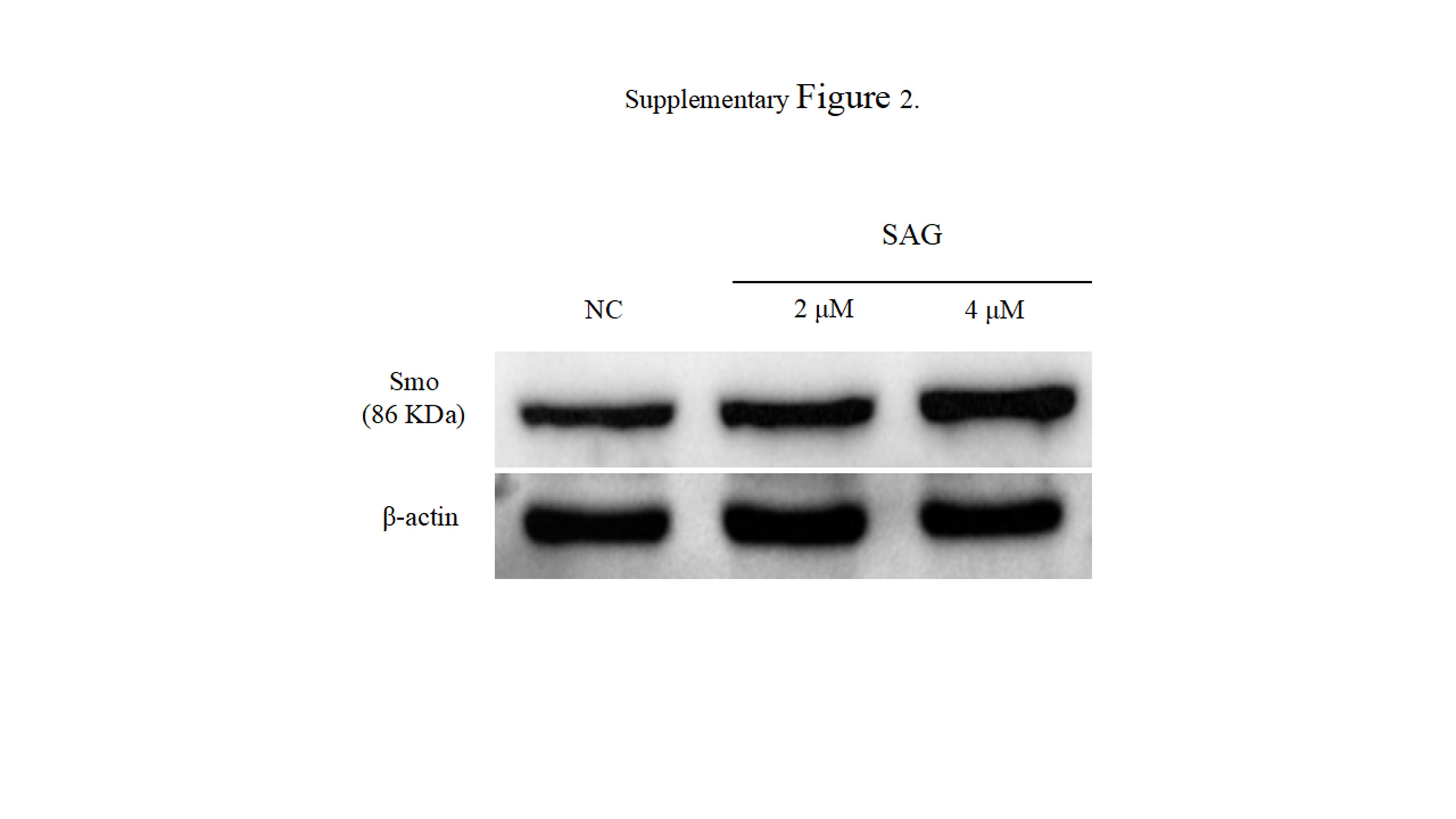

Supplement: Supplementary file 2 — Fig S2 [file JCMM-26-3213-s001.tif]
